# Structural, dynamical and symbolic observability: From dynamical systems to networks

**DOI:** 10.1371/journal.pone.0206180

**Published:** 2018-10-31

**Authors:** Luis A. Aguirre, Leonardo L. Portes, Christophe Letellier

**Affiliations:** 1 Programa de Pós-Graduação em Engenharia Elétrica, Universidade Federal de Minas Gerais, Belo Horizonte, Minas Gerais, Brazil; 2 School of Mathematics and Statistics, University of Western Australia, Perth, Western Australia, Australia; 3 Normandie Université — CORIA, Campus Universitaire du Madrillet, Madrillet, France; Universidad Rey Juan Carlos, SPAIN

## Abstract

Classical definitions of observability classify a system as either being observable or not. Observability has been recognized as an important feature to study complex networks, and as for dynamical systems the focus has been on determining conditions for a network to be observable. About twenty years ago continuous measures of observability for nonlinear dynamical systems started to be used. In this paper various aspects of observability that are established for dynamical systems will be investigated in the context of networks. In particular it will be discussed in which ways simple networks can be *ranked* in terms of observability using continuous measures of such a property. Also it is pointed out that the analysis of the network topology is typically not sufficient for observability purposes, since both the dynamics and the coupling of such nodes play a vital role. Some of the main ideas are illustrated by means of numerical simulations.

## 1 Introduction

One of the many concepts used to analyze dynamical systems and networks is observability. The genesis of this can be traced back to mid 20th century. It is interesting to see that depending on the research area observability has been painted with different colors. In control theory, the cradle of this concept [1], observability is related to the ability of reconstructing the state of the system from a limited set of measured variables in finite time. A somewhat relaxed version of this definition of observability and which is applicable to networks is known as structural observability and can be assessed with graphs [2]. These concepts have a main aspect in common: both classify the system as either being observable or not. In this paper the term *structural observability* will be used to refer to such a feature. In the case of networks such concepts could, in principle, be used to decide how many nodes should be measured in order to render a network observable.

There is a different approach to observability, which evolved from the traditional one, that has a different aim. Even if a system is observable, it might be advantageous, especially from a practical point of view, to measure specific variables. Instead of a crisp classification in terms of observability, this concept permits distinguishing between more and less observable scenarios [3, 4]. We shall refer to this as *dynamical observability*. Two decades ago, some of these concepts were adapted to rank variables of nonlinear dynamical systems based on observability [5] and from there appeared other related approaches that will be briefly reviewed in this work.

In the context of networks, the concepts of observability and its dual—controllability—have been recognized as relevant tools for analysis and design [6–10]. In this respect, two aspects stand out. First, classical procedures to determine if a system is observable face some serious practical and numerical difficulties when applied to larger systems. Indeed, it seems that in the case of high-dimensional networks, observability is more often than not investigated only from its topology (described by the adjacency matrix): this will be referred to as *topological observability* in this paper. As it will be shown, in the case of oscillators connected according to an adjacency matrix, investigating its connectivity, encoded by the corresponding graph, is typically not sufficient to assess the observability of the network. Second, to determine a minimum number of sensor nodes for which a network is observable is a valuable piece of information. But to be able to choose from alternative configurations is also an important practical problem that will receive attention in this work.

As it will be argued, the classical way of classifying systems as being observable or not—that is structural observability –, cannot really help much in solving the mentioned challenge as recently pointed out [9–11]. In order to do so, alternative scenarios of observable systems must be compared in order to decide which is more favorable. In other words, as it happened for dynamical systems, also for networks there should be a change in paradigm: from structural to dynamical observability.

The benefits and need for this have already been pointed out in the literature. For instance, it has been acknowledged that to choose variables that convey good observability of the dynamics enables estimating the state of a network of neuron models using Kalman-related methods [12, 13]. In a recent study about controllability and observability of network topologies built with neuron models, it has been found that “it is necessary to take the node dynamics into consideration when selecting the best driver (sensor) node to modulate (observe) the whole network activity” [8]. The reader should notice expressions such as “the need to pick *good* observables” or “to choose the *best* sensor nodes”. This type of challenge can be met conceptually using dynamical observability. Of course, the numerical challenge of determining such a property for a large network is of paramount importance and, at the moment, seems unsolved in general.

In view of all this, one of the aims of this paper is to review some concepts and procedures concerning observability in the context of nonlinear dynamics. It will be useful to see that observability can be classified into different types. Hopefully this classification will clarify the main differences which could help to answer some of the recent remarks that appeared in the literature. Also, the application of such concepts to networks will be discussed in the light of some classical and more recent methods for determining network observability. To this end, tools for nonlinear dynamics will be used. Even if from a numerical point of view, some of the used procedures are not feasible in the context of large networks, there is much to be gained in conceptual terms. In particular, numerical examples will be provided for showing that in investigating the observability of a dynamical network, both node dynamics and coupling must be considered. A simple example is provided to show that even linear oscillators connected according to the same adjacency matrix may result in either observable or unobservable networks depending on the variables used for coupling such oscillators.

### Terminology and organization

This paper shall refer to *dynamical networks* as the interconnection of dynamical systems. Such dynamical systems will sometimes be called oscillators and compose the node dynamics of the dynamical network. The interconnection of such nodes is according to a certain topology which is described by the adjacency matrix of the network. Graphs can be defined for: i) the node dynamics, which sometimes are referred to as fluence graphs; ii) the topology, and for iii) the full dynamical network (combining the node dynamics and the network topology). Only when the node dynamics are of first order, the graph of the network topology will coincide with the graph of the full dynamical network.

This paper is organized as follows. Section 2 reviews a number of concepts that underline the rest of the paper concerning observability, especially as they emerged from the field of dynamical systems. The counterpart, in the context of network topologies, is provided in Section 3. Different types or aspects of observability are then summarized in Section 4. Section 5 discusses the relevance of the aforementioned concepts in the case of nonlinear dynamical networks. That section also includes some simulation results. The main points are summarized in Section 6, where Table 1 is provided as a “road map” of this paper.

**Table 1:** Summary of types of observability and systems. Yes/No refers to practical applicability of numerical procedures discussed in the paper. The “Node dynamics” corresponds to low-dimensional dynamical systems interconnected according to a “Topology” to form a dynamical “Network”.

Type of Observability	Task	Node dynamics Sec. 2	Topology Sec. 3	Networks Sec. 5
Structural Sec. 4.1	observable vs. nonobservable classification	Yes Sec. 2.1	Yes Sec. 3.1–3.2	Yes
Symbolic Sec. 4.3	Ranking variables	Yes Sec. 2.5	Yes Sec. 3.4	Yes
Dynamical Sec. 4.2	Ranking variables	Yes Sec. 2.2–2.4	Only for small dimension Sec. 3.3	No

## 2 Observability of dynamical systems

The objective of this section is to give a brief historical background in order to set the remainder of the paper into context. The main ideas in this section are illustrated with examples of the paradigmatic Rössler system.

### 2.1 Either observable or not

The concepts of observability and controllability for linear systems are due to Rudolf Kalman [1]. Consider the linear system

$$\begin{array}{c} \{\begin{array}{ccc} \dot{x} & = & Ax+Bu\\ s & = & Cx, \end{array} \end{array}$$ (1)

where $x\in R^{n}$ is the state vector, $s\in R^{p}$ is the measurement vector, $u\in R^{r}$ is the input vector and (*A*, *B*, *C*) are constant matrices known respectively as the dynamics matrix, the input or control matrix and the output or measurement matrix. The system (1) is said to be observable at time *t*_*f*_ if the initial state ***x***(0) can be uniquely determined from knowledge of a finite time history of the output *s*(*τ*), 0 ≤ *τ* ≤ *t*_*f*_ [14] and the input *u*(*τ*) whenever it exists.

One way of testing whether the system (1) is observable is to define the *observability matrix*:

$$\begin{array}{c} O={\left[\begin{array}{ccccc} C & CA & CA^{2} & \ldots & CA^{n-1} \end{array}\right]}^{\text{T}}. \end{array}$$ (2)

The system (1) is therefore observable if matrix O is full rank, that is if its rank ρ[O]=n. This is known as Kalman’s rank condition for observability and according to it a pair [*A*, *C*] is either observable or not.

The concepts of controllability and observability were extended to nonlinear systems in the 1970s, e.g. [15]. Consider a nonlinear system

$$\begin{array}{c} \{\begin{array}{c} \dot{x}=f\left(x\right)\\ s\left(t\right)=h\left(x\right), \end{array} \end{array}$$ (3)

with $f:R^{n}\to R^{n}$ and, for simplicity s(t)∈R, that is $h:R^{n}\to R$. Differentiating *s*(*t*) yields

$$\begin{array}{c} \dot{s}\left(t\right)=\frac{d}{dt}h\left(x\right)=\frac{\partial h}{\partial x}\dot{x}=\frac{\partial h}{\partial x}\text{f}\left(x\right)=L_{f}h\left(x\right). \end{array}$$ (4)

$L_{f}h\left(x\right)$ is the Lie derivative of *h* along the vector field ***f*** and $s^{\left(j\right)}=L_{f}^{j}h\left(x\right)$. The observability matrix can be written as

$$\begin{array}{c} O_{s}\left(x\right)={\left[\begin{array}{ccc} \frac{\partial L_{f}^{0}h\left(x\right)}{\partial x} & \ldots & \frac{\partial L_{f}^{n-1}h\left(x\right)}{\partial x} \end{array}\right]}^{\text{T}} \end{array}$$ (5)

where the index *s* has been used to emphasize that $O_{s}\left(x\right)$ refers to the system observed from *s*(*t*).

The pair [***f***, *h*(***x***)] in (3) is said to be *observable* if $\rho \left[O_{s}\left(x\right)\right]=n,\;\forall x\in R^{n}$, which is the counterpart of Kalman’s rank condition for linear systems—see [15] for details. If [***f***, *h*(***x***)] is observable, any two initial conditions $x_{0_{1}}$ and $x_{0_{2}}$ are distinguishable with respect to the measured time series *s*(*t*), *t* ≥ 0.

Since observability is determined by a rank criterion in both cases, linear and nonlinear systems are classified either as observable or not.

An interesting step in the field was to recognize that the observability matrix in (5) is in fact the Jacobian matrix *of the map*

$$\begin{array}{c} \Phi_{s}\;:R^{n}\left(\text{x}\right)\mapsto R^{n}\left(s\left(t\right),s^{\left(1\right)},...,s^{\left(n-1\right)}\right), \end{array}$$ (6)

between the original and the *n*-dimensional differential embedding spaces [16]. If Φ_*s*_ is invertible (injective), it is possible to reconstruct the state from *s*(*t*). The condition for invertibility of Φ_*s*_ at ***x***_0_ is

$$\rho \left[\frac{\partial \Phi_{s}}{\partial x}|{}_{x=x_{0}}\right]=n.$$ (7)

Hence, the system is locally observable if condition (7) holds, that is, if Φ_*s*_ is locally invertible. If Φ_*s*_ is constant and invertible, then there is a global diffeomorphism and the pair [***f***, *h*] is fully observable. When the reconstructed space is *n*-dimensional, and thus $\frac{\partial \Phi_{s}}{\partial x}$ is a *n* × *n* matrix, it may be also useful to express condition (7) as [17] (see Example 1):

$$\begin{array}{c} \text{Det}\;\frac{\partial \Phi_{s}}{\partial x}\neq 0\;. \end{array}$$ (8)

**Remark 1**. If the dimension of the reconstructed space is allowed to increase using

$$\begin{array}{c} \Phi_{s}\;:R^{n}\left(x\right)\mapsto R^{d}\left(s\left(t\right),s^{\left(1\right)},...,s^{\left(d-1\right)}\right), \end{array}$$ (9)

with *d* > *n*, often, singularities that Φ_*s*_ may have will vanish and, then Φ_*s*_ gradually becomes full rank as would be expected from Takens’ theorem [18]. Relations between observability theory when more than one variable is measured and Takens’ theorem have been discussed in [19]. Increasing the *d* in order to remove singularities seems to have serious limitations when networks are considered [20].

**Example 1**. The Rössler system is [21]:

$$\begin{array}{c} \{\begin{array}{cc} \dot{x} & =-y-z\\ \dot{y} & =x+ay\\ \dot{z} & =b+z\left(x-c\right), \end{array} \end{array}$$ (10)

where (*a*, *b*, *c*) are parameters. If *s* = *y*, then the observability matrix is given by

$$\begin{array}{c} \frac{\partial \Phi_{y}^{3}}{\partial x}=O_{y}\left(x\right)=[\begin{array}{ccc} 0 & 1 & 0\\ 1 & a & 0\\ a & a^{2}-1 & -1 \end{array}], \end{array}$$ (11)

where $\Phi_{y}^{3}\;:R^{3}\left(x\right)\mapsto R^{3}\left(y\left(t\right),y^{\left(1\right)},y^{\left(2\right)}\right)$ and $O_{y}\left(x\right)$ is constant and nonsingular. Hence the Rössler system is observable from *y* at any point of the phase space.

### 2.2 Ranking observable pairs

Friedland defined the coefficient [3]

$$\begin{array}{c} \delta =\frac{|\lambda_{\text{min}}\left[O^{\text{T}}O\right]|}{|\lambda_{\text{max}}\left[O^{\text{T}}O\right]|}, \end{array}$$ (12)

where $\lambda_{\text{max}}\left[O^{\text{T}}O\right]$ indicates the maximum eigenvalue of $O^{\text{T}}O$ (likewise for λ_min_) for *linear* observability. Hence even for full row rank observability matrices, the observability coefficient 0 ≤ *δ* < 1 could be small, indicating “poor observability”. For a nonobservable pair [*A*, *C*], *δ* = 0. The following remarks are in order.

**Remark 2**. Ranking is of interest for *observable* pairs. Consider single-output linear systems, for which ${c\in R}^{n}$ and the output is ***s*** = ***c***^T^***x***. Hence we refer to the observability of the pair [*A*, **c**^T^]. Suppose two pairs $\left[A,c_{1}^{\text{T}}\right]$ and $\left[A,c_{2}^{\text{T}}\right]$ have observability matrices (see Eq 2) $O_{1}$ and $O_{2}$, respectively, such that $\rho \left[O_{1}\right]=\rho \left[O_{2}\right]=n$, therefore both systems are fully observable. Nevertheless, using (12) it is found that 0 < *δ*_1_ < *δ*_2_. In such a situation it is said that $\left[A,c_{1}^{\text{T}}\right]$ is less observable than $\left[A,c_{2}^{\text{T}}\right]$ or, alternatively, *s*_2_ ▷ *s*_1_ meaning that $s_{1}=c_{1}^{\text{T}}x$ (see Eq 1) provides worse observability of the dynamics in *A* than $s_{2}=c_{2}^{\text{T}}x$.

**Remark 3**. A similar result can be stated for nonlinear systems (***f***, *h*_1_) and (***f***, *h*_1_).

**Remark 4**. Hence, observability coefficients *δ* can be used to rank two pairs with $\Phi_{s_{1}}$ and $\Phi_{s_{2}}$, which are constant and invertible. This means that even if there are global diffeomorphisms, one situation could be preferable to the other. If the reconstructed space is *n*-dimensional, this can be directly assessed by the expression of Det $\frac{\partial \Phi_{s}}{\partial x}$ which can be nonzero but very small in the case of a poor observable.

An example of Remark 2 is provided by the theory of linear systems for which it is know that similarity transformations of coordinates do not change the rank of the observability or controllability matrices [14]. It was shown that *δ* in (12) and the counterpart controllability index *are* sensitive to similarity transformations because, although the rank of the grammian or observability or controllability matrices does not change, the numerical conditioning will generally different [4]. Similar findings in the context of networks have been reported by Sun and Motter. In particular it has been pointed out that even for fully controllable networks the practical implementation of controllers could be virtually impossible due to ill conditioning [22].

Following the ideas in [3, 4], the concept of ranking observable systems was adapted to nonlinear dynamical systems [5, 17]. In particular (12) was extended to:

$$\begin{array}{c} \delta_{s}\left(x\right)=\frac{|\lambda_{\text{min}}\left[O_{s}^{T}O_{s},x\left(t\right)\right]|}{|\lambda_{\text{max}}\left[O_{s}^{T}O_{s},x\left(t\right)\right]|}. \end{array}$$ (13)

The observability matrix $O_{s}\left(x\right)$ was originally evaluated using (2) with the Jacobian matrix D***f***(***x***) in place of the dynamics matrix *A*. In subsequent works, the observability matrix in Eq (5) was evaluated along a trajectory ***x***(*t*), *t*_0_ < *t* < *T* and index (13) averaged along ***x***(*t*), that is

$$\begin{array}{c} \delta_{s}=\frac{1}{T-t_{0}}\int_{t_{0}}^{T}\delta_{s}(x(\tau ))d\tau , \end{array}$$ (14)

where *T* is the final time considered and *t*_0_ > 0 is chosen to avoid the effect of transients. The observability coefficients are computed for the Rössler system in Example 2.

**Example 2**. For the Rössler system (10), the observability matrix from the *z* variable, $O_{z}\left(x\right)$, is

$$\begin{array}{c} \frac{\partial \Phi_{z}^{3}}{\partial x}=[\begin{array}{ccc} 0 & 0 & 1\\ z & 0 & x-c\\ b+2z(x-c) & -z & {\left(x-c\right)}^{2}-y-2z \end{array}], \end{array}$$ (15)

which is not constant. Because $\text{Det}(O_{z})=-z^{2}$ vanishes for *z* = 0 this system cannot be “seen” from the *z*-variable in the space $\left(z,\;\dot{z},\;\ddot{z}\right)$ when the original system is at ***x*** = [*x*, *y*, 0]^T^ which is the so-called singular observability manifold, [23]. Hence, $O_{z}\left(x\right)$ is rank deficient on the singular plane *z* = 0 and approximately rank deficient close to it. Using (11), $O_{x}\left(x\right)$ (not shown) and (15) in (13) and computing (14) the following values were found [16]: *δ*_*x*_ = 0.022, *δ*_*y*_ = 0.133 and *δ*_*z*_ = 0.006, hence the variables of the Rössler system can be ranked according to observability as *y* ▷ *x* ▷ *z*.

### 2.3 Singularities and lack of observability

Singularities in O(x) indicate that the map between the original state space and the considered reconstructed space is not globally invertible. As illustrated in Example 3, increasing *d* may eliminate such singularities, but *this is only the case for observable systems*. For nonobservable pairs, increasing *d* will not avoid singularities. This can be interpreted as a lack of genericity in the measurement function in terms of Takens’ theorem.

It will be convenient to distinguish between “local” and “global” singularities. A constant rank-deficient observability matrix O will be said to have a global singularity because it is always rank-deficient, regardless of where the system is in state space. This is always the case for nonobservable linear systems. On the other hand, for nonlinear systems O(x) may become rank-deficient at certain regions of state space. For instance, $O_{z}\left(x\right)$ in (15) becomes rank-deficient at *z* = 0. The existence of local singularities is a consequence of nonlinearity. A system with a global singularity in its observability matrix is nonobservable. This cannot be said of a system with an observability matrix with a local singularity.

Hence, observability can be affected by: i) the choice of coordinates of the reconstructed space, and ii) the existence of singularities and the way in which the trajectory relates to them. The first case can happen in linear systems [4] or nonlinear systems; the second case only happens in nonlinear systems.

**Example 3**. If the Rössler attractor is reconstructed in (*z*, *z*^(1)^, *z*^(2)^, *z*^(3)^), where *z*^(*i*)^ is the *i*th derivative of *z*, the corresponding map is $\Phi_{z}^{4}$, where the superscript 4 indicates the dimension of the reconstructed space. Therefore, the observability matrix for *z* = 0 (which in Example 2 has been shown to be rank deficient on the singular plane in the 3D reconstructed space) becomes [19]:

$${\frac{\partial \Phi_{z}^{4}}{\partial x}|}_{z=0}\begin{array}{c} =\left[\begin{array}{ccc} x-0 & 0 & 1\\ 0 & 0 & x-c\\ b & 0 & -y+{\left(x-c\right)}^{2}\\ 2b\left(x-c\right) & -2b & \phi_{4,3} \end{array}\right], \end{array}$$ (16)

where *ϕ*_4,3_ = −3*b* − *x* − *ay* + (*x* − *c*)[−3*y* + (*x* − *c*)^2^] and which is a full column rank matrix.

Hence there is an embedding from $R^{3}\left(x,y,z\right)$ to ${\mathbb{R}}^{4}\left(z,z^{\left(1\right)},z^{\left(2\right)},z^{\left(3\right)}\right)$ and that the system is observable from such a reconstructed space. Alternatively, it can be said that there is a global diffeomorphism from the *attractor* in $R^{3}\left(x,y,z\right)$ to the one in ${\mathbb{R}}^{4}\left(z,z^{\left(1\right)},z^{\left(2\right)},z^{\left(3\right)}\right)$—both attractors have the same dimension. Nonetheless, this was attained at the expense of increasing the dimension of the reconstructed *space*. This was not required for the *y* variable. Hence *y* provides a more favorable situation than *z* as quantified by the observability coefficients.

### 2.4 Graphical approaches

Convenient ways of assessing and interpreting observability can be developed using graphical techniques. In [24] a procedure was put forward that does not result in numerical indices, it falls into the category of ranking observable systems. This is an important point because, as it will be seen later in Sec. 3, there are other graphical procedures that follow the *either observable or not* framework.

The method in [24] consists of representing the variables of *a single* dynamical system and the corresponding relationship by means of a graph that resembles an inference diagram. In such a diagram, linear and nonlinear dependencies are indicated by continuous and dashed arrows, respectively, as shown in the next example.

**Fig 1:**
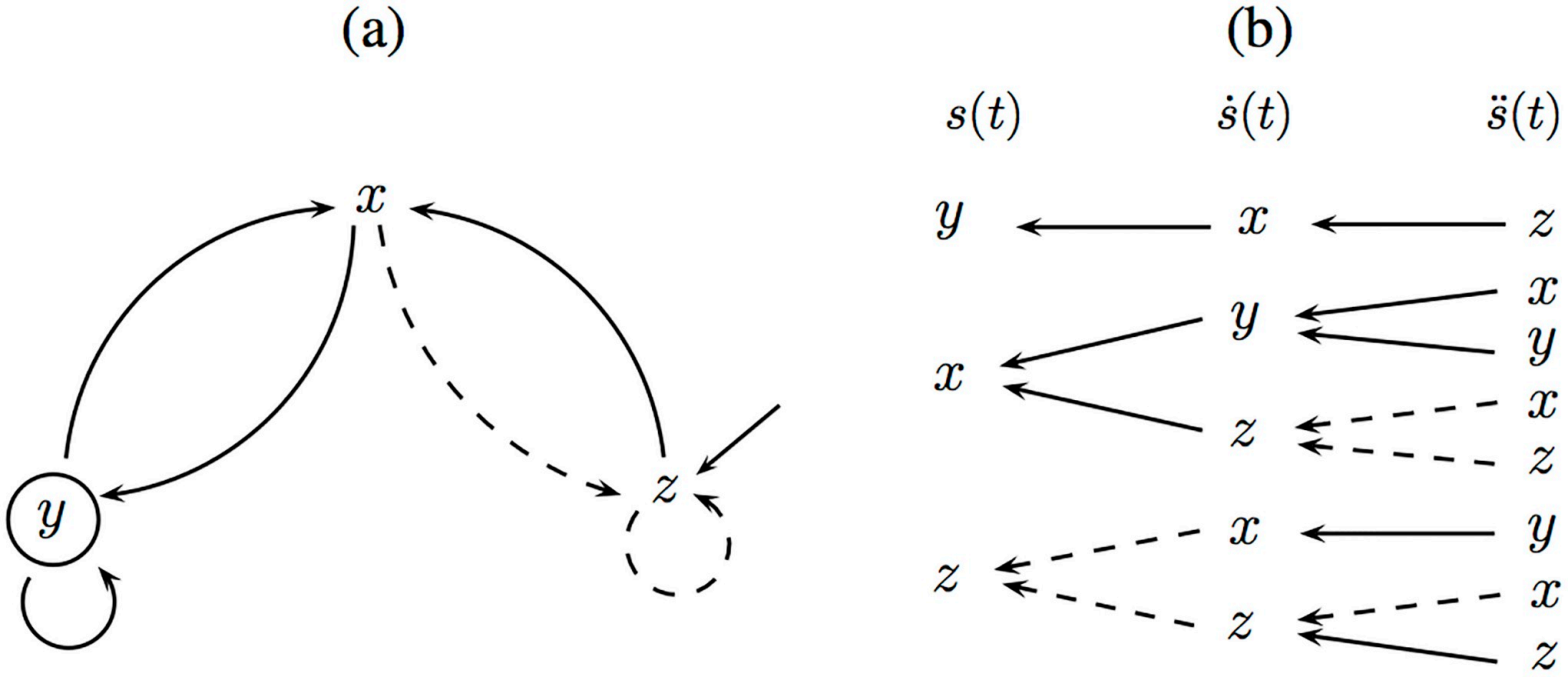
Graphical observability analysis for the Rössler system. (a) Graph of the interaction between the variables for the Rössler system. A solid (dashed) arrow represents a (non) linear coupling. (b) Unfolded schematic view of the variables reached when the first and the second derivative are computed.

**Example 4**. The first equation of (10) tells us that variables *y* and *z* act linearly on *x*. Thus, arrows from vertices *y* and *z* will reach *x* with a solid line. The second and third equations can be interpreted likewise. The whole graph is shown in Fig 1a. The solid arrow pointing to *z* represents the constant *b*. This graph is drawn from the Eq (10), regardless of the recorded variable *s*(*t*).

From the graph in Fig 1a, an unfolded scheme (Fig 1b) is built by graphically assuming that each node variable is measured one at a time, that is, *s*(*t*) = *x*, *y*, *z*. This is done in order to highlight the differences among such variables in what concerns observability. To this end, the unfolded scheme is obtained by visiting the variables starting from the measured variable *s*(*t*), and moving against the arrow directions. Each column in Fig 1b corresponds to an additional dimension in embedding space. For instance, when *y*(*t*) is measured—hence *s*(*t*) = *y*(*t*)—it serves as the first coordinate. The second and third coordinates are $\dot{y}$ and $\ddot{y}$, respectively. Starting from node *y* (first coordinate), we move one step agains the arrow to produce $\dot{y}$ (the second coordinate). Since the arrow is continuous and ends at node *x* in Fig 1a, a continuous connection appears in Fig 1b between *y* and *x*, and so on.

Since continuous arrows indicate linear relationships, whenever the three variables (*x*, *y*, *z*) are connected horizontally by solid arrows there is a global diffeomorphism. This is because the links will not vanish and the 3D space has information about all the coordinates of the original space. Hence in Fig 1b it is seen that the only global diffeomorphism happens between the original state space and $\left(y,\;\dot{y},\;\ddot{y}\right)$. Contrary to this, dashed arrows arise from nonlinear interactions that are related to singularities in the map from the original to the embedding space. The sooner dashed arrows appear in the unfolded scheme the worse from an observability point of view. Hence, when *x* is measured dashed arrows appear in the last stage, connecting $\dot{x}$ to $\ddot{x}$, whereas for the *z* variable, there are dashed arrows already in the first stage. As a consequence, *z* provides worse observability of the system dynamics than *x*, hence *y* ▷ *x* ▷ *z*.

### 2.5 Symbolic observability

As discussed in Sec. 2.3, one of the aspects that greatly influence observability in nonlinear systems are the singularities that appear in the observability matrix. Because at a singularity the determinant of the *n* × *n* observability matrix will become null, the underlying motivation in symbolic observability is that the more complicated the determinant $\text{Det}[{\tilde{O}}_{s}$] of the symbolic observability matrix, the less observable the system is [25].

The computation of $\text{Det}[{\tilde{O}}_{s}$] can be a nearly impossible task for five-dimensional rational system. Nevertheless the complexity of $\text{Det}[{\tilde{O}}_{s}$] can be assessed simply by counting the number of linear, nonlinear and rational terms in it, without paying attention to its exact form and this will suffice to quantify observability [26].

The main steps for computing symbolic observability indices are: i) obtain the symbolic Jacobian matrix $\tilde{J}$ from the classical Jacobian matrix by replacing constant, non-constant polynomial, and rational elements, respectively with 1, $\bar{1}$, and $\bar{\bar{1}}$ (see Example 5); ii) build the symbolic observability matrix ${\tilde{O}}_{s}$ as detailed in [26], iii) compute the symbolic expression for $\text{Det}[{\tilde{O}}_{s}$] and count the number of symbolic terms in such an expression, iv) finally, the symbolic observability coefficient is obtained as

$$\begin{array}{c} \begin{array}{cc} \eta_{s^{n}}= & \frac{N_{1}}{N_{1}+N_{\bar{1}}+N_{\bar{\bar{1}}}}+\frac{N_{\bar{1}}}{{\left(\text{max}\left(N_{1},1\right)+N_{\bar{1}}+N_{\bar{\bar{1}}}\right)}^{2}}\\ & +\frac{N_{\bar{\bar{1}}}}{{\left(\text{max}\left(N_{1},1\right)+N_{\bar{1}}+N_{\bar{\bar{1}}}\right)}^{3}}, \end{array} \end{array}$$ (17)

where *N*_1_, $N_{\bar{1}}$ and $N_{\bar{\bar{1}}}$ are the numbers of symbolic terms 1, $\bar{1}$ and $\bar{\bar{1}}$, respectively. In the symbolic approach, the known equations are used to check whether the elements of the Jacobian matrix of the system are constant, non-constant polynomial or rational. When investigating a network with nodes that are copies of the same dynamics, this can be treated in an automatic way from the Jacobian matrix of the node dynamics and the adjacency matrix. What can be computationally long is to test all possible combinations between the measured variables and their retained derivatives. The number of these possibilites can be significantly reduced by either investigating the symbolic observability matrix [11] or by using a graphical approach [29].

**Example 5**. For the Rössler system (10), the Jacobian and symbolic Jacobian matrices are

$$\begin{array}{c} D\text{f}=[\begin{array}{ccc} 0 & -1 & -1\\ 1 & a & 0\\ z & 0 & \left(x-c\right) \end{array}];\tilde{J}=[\begin{array}{ccc} 0 & 1 & 1\\ 1 & 1 & 0\\ \bar{1} & 0 & \bar{1} \end{array}]\;, \end{array}$$ (18)

respectively. Notice that $\tilde{J}$ can be obtained from D***f*** by inspection. If variable *x* is measured, the respective observability matrix is given by [26]:

$$\begin{array}{c} {\tilde{O}}_{x}=[\begin{array}{ccc} 1 & 0 & 0\\ 0 & 1 & 1\\ \bar{1} & 1 & \bar{1} \end{array}], \end{array}$$ (19)

for which the symbolic determinant is $\text{Det}[{\tilde{O}}_{x}]=1⊗\left(1⊗\bar{1}-1⊗1\right)$. In that expression there are four 1s, and one $\bar{1}$, hence *N*_1_ = 4, $N_{\bar{1}}=1$ and $N_{\bar{\bar{1}}}=0$. Using these values in (17) yields *η*_*x*^3^_ = 0.84. Similarly [26]: *η*_*y*^3^_ = 1 and *η*_*z*^3^_ = 0.56, where the exponent indicates the dimension of the reconstruction space (see Example 3). Therefore the variables can be ranked as before *y* ▷ *x* ▷ *z*.

### 2.6 Data-based observability

All the types of observability discussed so far are defined based on the system equations. Motivated by the fact that in practice the system equations are not always available, an alternative procedure for assessing observability was proposed in [27]. However, observability is, by definition, related to the equations of the vector field or related to the map, in the case of discrete-time systems. Hence estimating coefficients from data is only an indirect way of assessing observability from some of its *signatures* found in a reconstructed space.

## 3 Graphical approaches for assessing observability

This section is devoted to graph-theoretic approaches for assessing observability of dynamical systems. When a network is considered, there are three levels of description: i) the node dynamics, commonly made of a dynamical system (oscillator), ii) the topology of the network, described by the corresponding adjacency matrix, and iii) the full network combining the node dynamics with the network topology. Each level can be represented by a specific graph providing different assessment of the network observability as it will be addressed in Sec. 5. Given the importance of graphs, this section reviews some results concerning the quantification of observability from such a representation. Some examples will be taken using simple dynamical systems (oscillators).

### 3.1 Lin’s method

In a seminal paper, Lin developed the concept of structural controllability [2] which was later extended to that of structural observability in [28]. Such concepts have been defined for linear systems as (1). In words, a linear dynamical pair [*A*, *C*] is structurally observable if there exists a “perturbed” pair [*A*_1_, *C*_1_] of the same dimension with the same structure which is completely observable. [*A*, *C*] and [*A*_1_, *C*_1_] are of the same structure if for every fixed zero entry of [*A*, *C*] the corresponding entry of the pair [*A*_1_, *C*_1_] is also a fixed zero and vice-versa [28]. Also, [*A*_1_, *C*_1_] is a perturbed pair of [*A*, *C*] in the sense that there exists an *ϵ* > 0 such that ||*A* − *A*_1_|| < *ϵ* and ∥*C* − *C*_1_ ∥ < *ϵ*. For instance, consider the pair

$$\begin{array}{c} A=[\begin{array}{cc} A_{11} & 0\\ A_{21} & A_{22} \end{array}],\;C=[\begin{array}{cc} C_{1} & 0 \end{array}], \end{array}$$ (20)

where the nonzero entries can assume any values. Clearly, the observability matrix (2) will be rank deficient regardless of the values of *A*_*ij*_ and of *C*_1_, hence the pair (20) is (structurally) nonobservable.

A very interesting analysis proposed by Lin was the drawing of a graph for the pair [*A*, ***b***]. An extension of Lin’s procedure for the case of observability can be easily accomplished by means of the *duality theorem* [14] by which the pair [*A*, ***c***^T^] (see Remark 2.2) is structurally observable iff its dual [*A*^T^, ***c***] is structurally controllable. When matrix *A* is transposed, the arrows of the edges should point in the reverse direction.

**Example 6**. In this example it is shown how the Rössler system (10) can be represented using a graph such that a procedure akin to Lin’s can be followed. Lin’s starting point is the dynamic matrix *A* and the input vector ***b***. The controllability of the Rössler system can be investigated using the Jacobian matrix D***f*** of (10) and ***b***:

$$\begin{array}{cc} & \begin{array}{ccccccc} \;x & \;y\; & \;\;z & \;b_{x} & \;\;\vdots & \;b_{y}\; & \;b_{z} \end{array}\\ & \left[D\text{f}\;\vdots \;b\right]=[\begin{array}{ccccccc} 0 & -1 & -1 & 1 & \vdots & 0 & 0\\ 1 & a & 0 & 0 & \vdots & 1 & 0\\ z & 0 & x-c & 0 & \vdots & 0 & 1 \end{array}]\;\begin{array}{c} \to x\\ \to y\\ \to z \end{array} \end{array}$$ (21)

**Fig 2:**
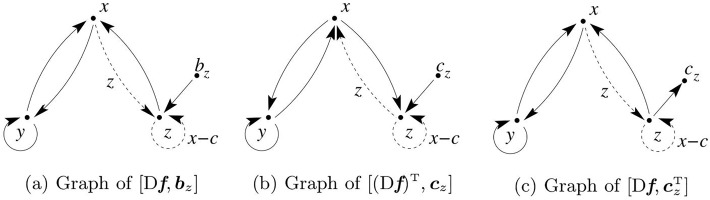
Graphs for controllability and observability analysis. (a) Graph of the pair D***f***, ***b***_*z*_] where ***b***_*z*_ = [0 0 1]^T^ is the input vector. (b) Graph of [(D***f***)^T^, ***c***_*z*_], the dual of (a), where $c_{z}^{\text{T}}=\left[0\;0\;1\right]$ is the input vector. (c) Graph of [D***f***, ***c***_*z*_] used with the “dual interpretation”. Dashed lines indicate non constant connections due to nonlinearities. Notice the similarity with the graph in Fig 1a.

Fig 2a shows the graph of pair [D***f***, [0 0 1]^T^]. Vertices *x* and *y* are both accessible from vertex *b*_*z*_: the Rössler system is structurally controllable when the system is driven from the *b*_*z*_ vertex. When the control is applied to variable *y*, vertex *x* is accessible but vertex *z* will not be accessible if the dashed link vanishes (*z* = 0): the pair [D***f***, [0 1 0]^T^] is therefore not structurally controllable for *z* = 0. A similar result is obtained for the pair [D***f***, [1 0 0]^T^].

In order to investigate the observability using Lin’s result, we have to use the dual system (Fig 2b). At *z* = 0 the connection from vertex *z* to vertex *x* vanishes and both *x* and *y* become non-accessible vertices (Fig 2b). Hence at *z* = 0 the pair (D***f***)^T^, ***c***] is noncontrollable and, from the duality theorem, this implies that[D***f***, ***c***^T^] is not observable at *z* = 0, as seen in Example 2.

We can reach a similar conclusion from the graph in Fig 2a but drawing an output vector ***c*** (Fig 2c) and using a “dual interpretation” for the edges. Thus, an edge from *v*_*i*_ to *v*_*j*_ means that *v*_*j*_ receives information from *v*_*i*_. Fig 2c illustrates the case when *z* is measured. Because the flow of information from *x*—and consequently from *y*—is cut when *z* = 0, the pair [D***f***, [0 0 1]] is structurally nonobservable. In this way, it is found that the pair [D***f***, [0 1 0]] is structurally observable. This is in agreement with the fact there exists a global diffeomorphism between the original state space and $\left(y,\dot{y},\ddot{y}\right)$ [16].

From the discussion above, it is clear that structural observability is unable to distinguish, given an *observable* system, situations with different observability features. For instance, for 0 < *z* ≪ 1 the edge linking *z* to *x* in Fig 2 has not yet vanished and the system remains structurally observable as well as for another system for which such a link has a constant weight. This weakness of addressing the observability of a graph is overcome by other definitions of observability.

As a consequence of nonlinearity there will be non constant elements in [(D***f***)*^T^* ⋮ **c**] and therefore there will be dashed connections (that can vanish) in the graph. Hence procedures to investigate observability that treat constant and variable connections alike ignore the effect of nonlinearity which is one of the main causes of singularities which, in turn, greatly affect the observability of a system, as discussed in Sec. 2.3.

### 3.2 Liu and coworkers’ method: Sensor sets

A more recent procedure has been put forward by Liu and coworkers who have addressed the problem of determining the minimum number of sensor nodes needed to reconstruct the state [6]. First, an inference diagram is built, this is a graph. The graph is decomposed in strongly connected components (SCC) which are the largest subgraphs in which there is a directed path from every vertex to any other vertex. If an SCC does not have any incoming edges, it has been called a root SCC [6]. Observability of the whole system is claimed to be achieved if at least one vertex of each root SCC is measured.

**Fig 3:**
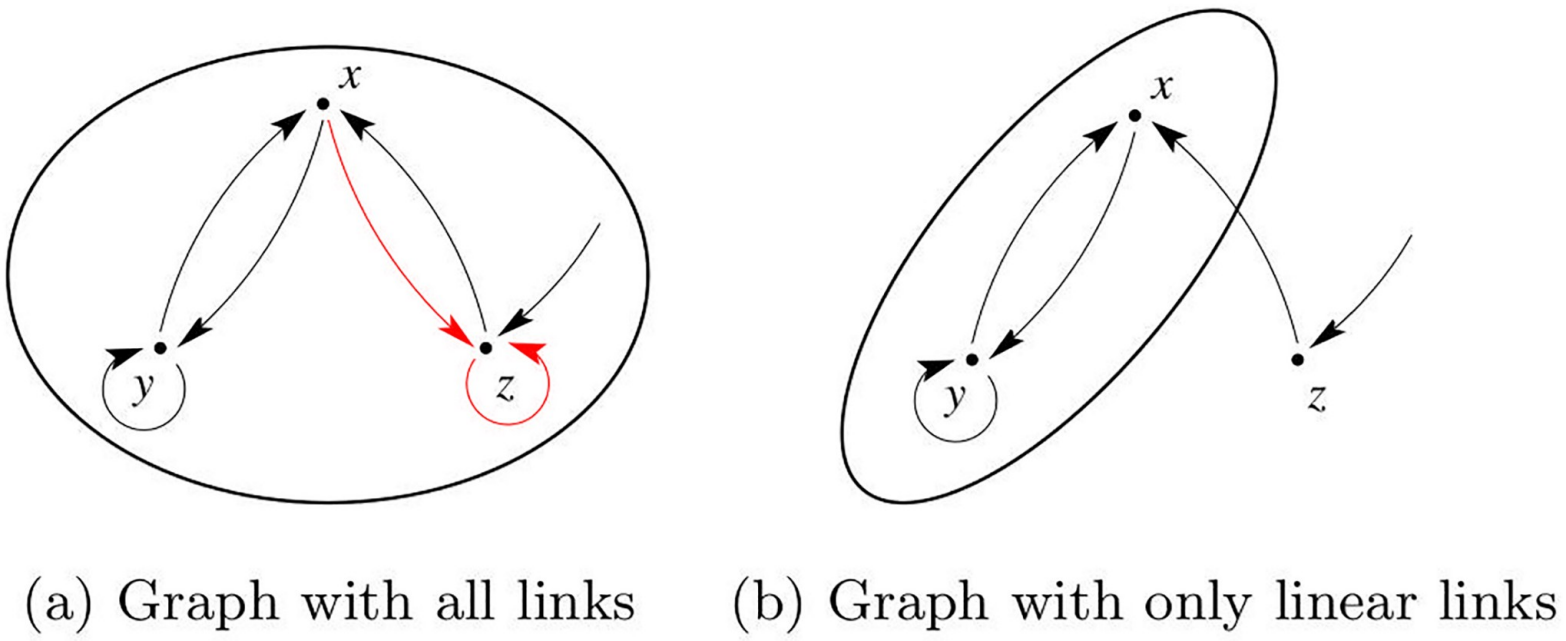
Graphs of the Rössler system. The root SCC (drawn as a thick circle) contains the three variables when the method considers all connections constant (a) and only variables *x* and *y* when nonlinearities are removed for building the graph (b).

**Example 7**. We start with the graph shown in Fig 1a which corresponds to the Rössler system (10) but without distinguishing between full and dashed lines. Notice that it is possible to start at any vertex (node or variable) and reach all other vertices following the arrows. Hence, the whole graph is an SCC. Because there is no incoming edge, this is also a root SCC. Hence in order to guarantee observability it suffices to measure any of its variables. However, if the dashed line vanishes, the *z* variable will no longer be part of the SCC (see Fig 3b) and should not be measured.

Example 7 shows that this method, as acknowledged by the authors [6, p. 2464] is unable to indicate that measuring the *y* variable from the Rössler system is preferable to, say, measuring *z*. On the other hand, it was shown that this graphical approach underestimates the number of variables which must be necessarily measured [10, 11]. An improved version of this graphical approach was recently proposed [29], showing that nonlinear interactions should be removed for determining the root SCCs and that such graph only provides necessary but not sufficient conditions on the measurements for ensuring structural observability.

### 3.3 Ranking observable graphs

In Lin’s method for structural observability (Sec. 3.1) only the presence or absence of edges is of concern. Therefore the method either classifies the graph as observable or not.

A more challenging situation is furnished by the pair $\left[A,\;\tilde{C}\right]$ with *A* given in (20) and $\tilde{C}=\left[C_{1}\;C_{2}\right]$, as follows. If *C*_2_ ≠ 0 the pair stands a chance of being observable. Let us assume that it is observable, that is, the observability matrix (2) computed with the pair $\left[A,\tilde{C}\right]$ is full rank,. Structural observability will be lost only if *C*_2_ = 0, and even for extremely small values of *C*_2_, the pair will be structurally observable. Hence such type of observability will not distinguish among a whole range of pairs that can be either far or arbitrarily close to the condition *C*_2_ = 0. A possible way out in this very simple example is to compute the condition number (12) for the observability matrices of $\left[A,\tilde{C}\right]$ for the different measuring situations that result in different $\tilde{C}\text{s}$. Ill-conditioned observability matrices will indicate unfavorable situations in terms of observability.

As for the method by Liu and coworkers for sensor set selection, the lack of discriminatory power pointed out in Example 7 is due to disregarding the differences in the type of edges, that is, the method treats full and dashed arrows alike. In order to rank the variables, features of the links should be taken into account, such as the weight of a link: small constant weights might result in ill-conditioned observability matrices and variable weights will give rise to singularities in such matrices. Hence such features will usually give rise to poorly observed regions and must be taken into account.

As it happened in the development of the theory of observability for dynamical systems, the first results classified graphs either as being observable or not. It seems that it would be desirable to see the development of procedures to rank graphs in terms of observability.

### 3.4 Symbolic observability of topologies

Provided that the symbolic Jacobian matrix J can be written for a graph then, in principle, symbolic observability coefficients can be computed. For relatively simple systems, to obtain J is straightforward, as the following example shows.

**Example 8**. We again consider the graph shown in Fig 1a. In a typical graph, there would be no distinction between full and dashed lines, as for the methods of Lin and of Liu and coworkers. Calling $J^{0}$ a symbolic Jacobian matrix that does *not* take into account the nonlinear connections, and $\tilde{J}$ the standard symbolic Jacobian matrix [26], from system (10) we get

$$\begin{array}{c} J^{0}=[\begin{array}{ccc} 0 & 1 & 1\\ 1 & 1 & 0\\ 1 & 0 & 1 \end{array}]\;\text{and}\;\tilde{J}=[\begin{array}{ccc} 0 & 1 & 1\\ 1 & 1 & 0\\ \bar{1} & 0 & \bar{1} \end{array}]. \end{array}$$ (22)

Notice that $\tilde{J}$ is the same as obtained in (18). Hence proceeding as in Example 5 the same symbolic observability coefficients obtained from the system equations are found using $\tilde{J}$, that is, from the graph. If $J^{0}$ is used instead, the result reached at is that any of the variables provide the same level of observability. This shows why the method by Liu and co-workers is unable to provide guidance of which sensor vertex to use within the root SCC which here (Fig 3a) contains the three variables. In the spirit of symbolic coefficients, the modified approach [29] does not take into account the nonlinear edges (Fig 3b).

For graphs of even moderate sizes, it might not be feasible to build analytical observability matrices. A software like Maple fails to compute the observability matrix of a 5D rational system [11]. Symbolic approaches are therefore an alternative to overcome this difficulty.

## 4 Types of observability

The aim of this section is to recognize differences among types of observability in what concerns definitions and aims, as reviewed in sections 2 and 3. Links between definitions will be pointed out and some extensions to networks will be proposed. The main results are summarized in Table 1.

### 4.1 Structural observability

The adjective *structural* was used by [2] to indicate cases in which controllability was robust against perturbations of unknown parameters. Here we use *structural* in a somewhat wider, but closely related, sense. Definitions of observability that classify a system in either observable or not are included in the class of *structural observability*. The justification for this is that in such cases, observability only depends on the internal structure (presence and nature of coupling terms) of the system variables. In this sense, Kalman’s definition and the nonlinear counterpart [15] belong to this class although such are sometimes referred to as being definitions of *complete* or *full* observability. Other terms such as *exact* and *mathematical* controllability/observability have been used recently [30].

A slightly different aim has been pursued in [6] where a minimum set of sensor vertices is sought in order to render a graph observable or not observable.

The aspect common to all such procedures reviewed in sections 2.1, 3.1 and 3.2 is a classification of a system according to which it is either observable or not.

### 4.2 Dynamical observability

In contrast to structural observability, we shall refer to *dynamical observability* whenever there is a continuous quantification of our ability to estimate the state of a system from a finite set of data. This can be done computing observability coefficients as discussed in sections 2.2 and 2.3. This class of observability only makes sense for systems that *are* observable. Hence dynamical observability helps us to rank *observable* pairs [***f***, *h_i_*(***x***)] for a given vector field ***f***.

A similar situation in terms of controllability of linear complex networks has been reported, namely the situation in which a network is controllable however, in practice, control is very difficult to attain [30]. As argued by Cowan and coworkers: “more important than issues of structural controllability are the questions of whether a system is almost uncontrollable” [31]. This is the typical situation in which a *dynamical* rather than a *structural* assessment of controllability or observability is called for. Dynamical observability was investigated in the context of three-node networks of Fitzhugh-Nagumo oscillators in [7].

In assessing this type of observability, there are two challenges to be faced. First is how to quantify how far the system is, at a certain point, from the location in space where observability is lost, that is, where observability matrix becomes rank deficient. Second, how to average this result in order to have a single “global” indication of observability. In Sec. 2.2 these challenges were met by computing the condition number (13), and taking an average along a trajectory (14) which can be interpreted as a spatial average in state space.

Other ways of facing the first challenge would be to use the determinant of the observability matrix or its singular values. The fraction of time that the trajectory spends within a neighborhood of the singularity manifold has been used to assess dynamical observability [23].

The coefficients that quantify dynamical observability have only relative interpretation and are not comparable in general among different systems. This shortcoming is overcome by the coefficients for symbolic observability, as discussed in Sec. 2.5.

### 4.3 Symbolic observability

*Symbolic observability* shares some features of the previous types of observability and includes characteristics of its own. On the one hand, as with structural observability, symbolic observability does not depend on parameter values but only on the nonlinear couplings within the system variables. On the other hand, as with dynamical observability, symbolic observability is capable of ranking observable pairs.

Central to the definition of symbolic observability is the complexity of the singularities that appear in the symbolic observability matrix. Some advantages compared to the other definitions are the fact that it is more amenable to be computed for larger systems with more complicated dynamics [26], it provides “normalized” results in the range [0; 1] that permit comparing different systems in terms of observability. Related to this, it has been argued that systems with a symbolic observability coefficient greater than 0.75 have good overal observability properties [32].

These symbolic coefficients are very promising for assessing the observability of systems and networks that are larger than the ones analyzed with the dynamical observability coefficients [11].

## 5 Observability of dynamical networks: Numerical results

A dynamical network is a set of dynamical systems—oscillators—interconnected according to the network topology which is described by the corresponding adjacency matrix. The aim is to discuss, in the context of a simple example where the node dynamics is linear, some of the aspects seen so far.

Here we will consider a network whose topology is described by the adjacency matrix

$$\begin{array}{c} A_{\text{adj}}=[\begin{array}{ccc} 0 & a_{12} & 0\\ a_{21} & 0 & a_{23}\\ 0 & a_{32} & 0 \end{array}], \end{array}$$ (23)

and for which at each node there is a three-dimensional dynamical system

$$\begin{array}{c} S_{i}:{\left({\dot{x}}_{i},{\dot{y}}_{i},{\dot{z}}_{i}\right)}^{\text{T}}={\left(-y_{i},x_{i},\alpha x_{i}-z_{i}\right)}^{\text{T}}. \end{array}$$ (24)

Nodes are coupled via one of their variables (*x*_*i*_, *y*_*i*_ or *z*_*i*_). In this network, the term *a*_32_ may vanish, for instance due to a nonlinear coupling. In what follows, we adopt the convention that the element *a*_*ij*_ of the adjacency matrix *A*_adj_ corresponds to an edge from vertex *j* to vertex *i* [33, Sec. 6.2]. If the other convention were adopted, we would have to use $A_{\text{adj}}^{\text{T}}$ in place of matrix *A* or the Jacobian matrix.

**Fig 4:**
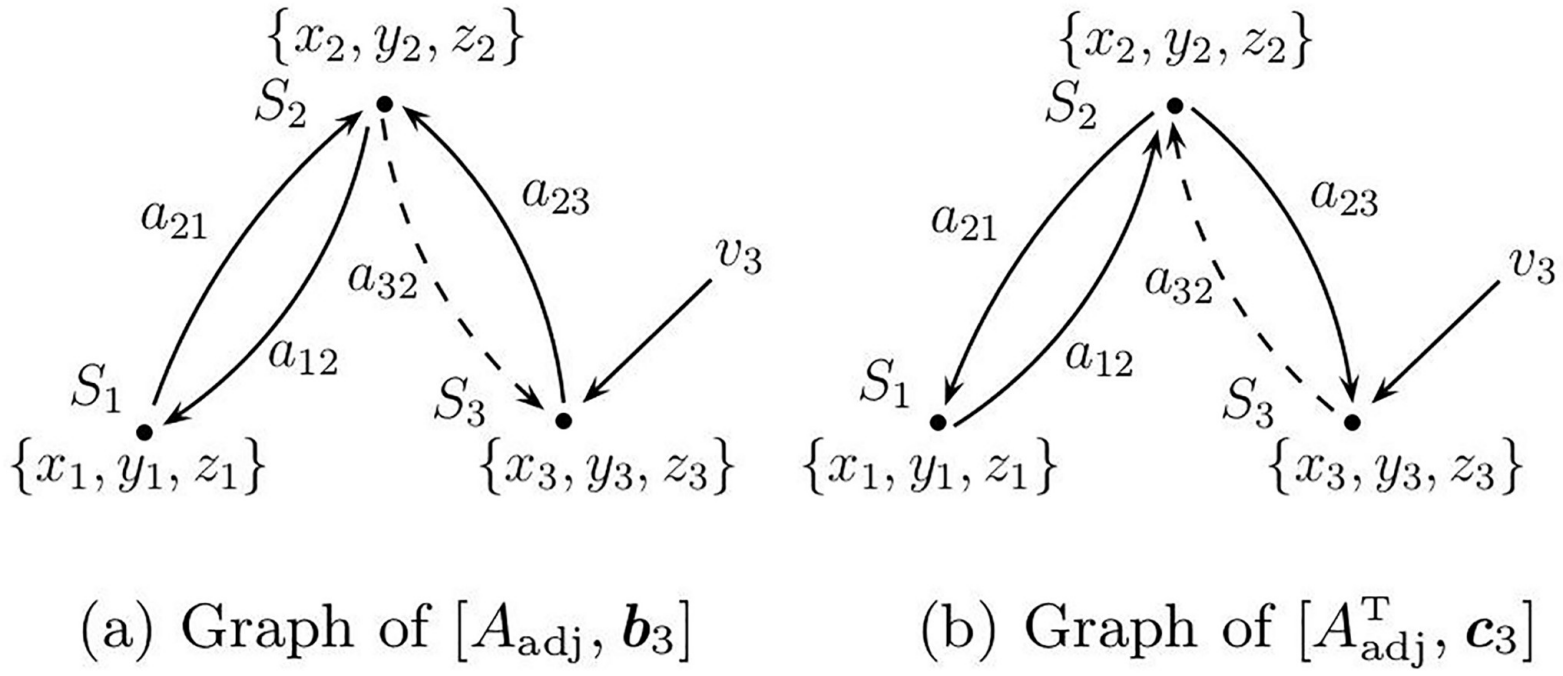
Graphs based on topology described by the adjacency matrix. (a) Graph of the network whose topology is described by the adjacency matrix (23) with a single driving node *S*_3_, and (b) the dual graph. If *a*_32_ vanishes in (b), nodes *S*_1_ and *S*_2_ become non-accessible and the dual network is no longer structurally controllable, hence the pair $\left[A_{\text{adj}},{\text{c}}_{3}^{\text{T}}\right]$ is no longer structurally observable from *S*_3_.

Consequently, following Newman’s convention, controllability can be investigated by considering the pair [*A*_adj_, ***b***] where ***b*** = [0 0 1]^T^, hence only system *S*_3_ receives the driving signal (Fig 4). As long as *a*_32_ ≠ 0 the network is structurally *topologically* controllable since each node can be reached from vertex *v*_3_ (Fig 4a). The *topological* observability of the network can be analyzed using the dual pair $\left[A_{\text{adj}}^{\text{T}},c\right]$ where ***c*** = [0 0 1]^T^, hence only one or more variables from *S*_3_ can be measured (Fig 4b). As long as *a*_32_ ≠ 0 the network is structurally topologically observable.

Using symbolic observability and treating the adjacency matrix *A*_adj_ as a Jacobian matrix, it is readily found that the network in Fig 4a is not topologically observable from *S*_2_ ($\eta_{2}^{3}=0$), it is fully topologically observable from *S*_1_ ($\eta_{1}^{3}=1$) and is poorly topologically observable from *S*_3_ ($\eta_{3}^{3}=0.56$). The lack of observability from *S*_2_, which can be readily confirmed from linear system theory, is not obvious, as this node receives information from the other two nodes. This result seems to be in line with the discussion presented in [9].

**Fig 5:**
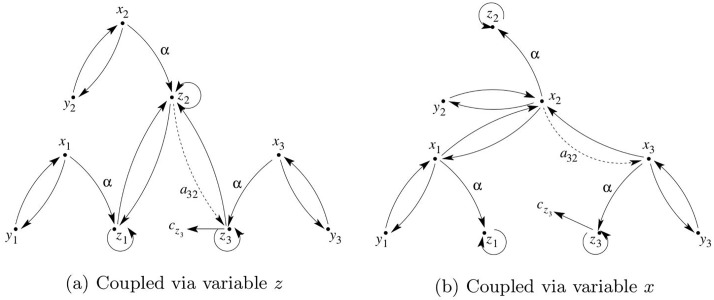
Graph of the full network with the topology in Fig 4a. (a) Coupled via variable *z*. (b) Coupled via variable *x* The details of the node dynamics are included. If *z*_3_ is measured, and *α* = 0 the network is not structurally observable regardless of the value of *a*_32_.

When considering the observability of a dynamical network as shown in Fig 4a with nodal dynamics (e.g. as given in Eq 24), it must be realized that the topological observability only provides a partial answer. In order to ensure structural observability of the full network from, say, *v*_3_, not only every *node* of the dual pair $\left[A_{\text{adj}}^{\text{T}},{\left[0\;0\;1\right]}^{\text{T}}\right]$ (Fig 4b) must be accessible by acting on *v*_3_ but also every *vertex* of the full network as shown in Fig 5a. In fact, the result strongly depends on the observability conveyed by the variable used in measuring the sensor node *and* the one used for coupling the nodes.

Since (24) is linear, it is straightforward to verify that the pair $\left[D{\text{f}}_{3},c_{3}^{\text{T}}\right]$ is structurally observable only if the measured variable is *z*_3_ (that is, $c_{3}^{\text{T}}=\left[0\;0\;1\right]$) and *α* ≠ 0. Therefore, although the network is structurally *topologically* observable from *S*_3_ (*a*_32_ ≠ 0), it is only structurally observable if variable *z*_3_ is recorded at *S*_3_. In addition, if *α* = 0, the network is *not* structurally observable even for *a*_32_ ≠ 0. Indeed, in Fig 5a the node dynamics, *S*_3_, is structurally observable when variable *z*_3_ is measured ($\eta_{z_{3}^{3}}=1$, Det $\frac{\partial \Phi }{\partial x}=-\alpha^{2}$ where $\Phi \;:\left(x_{3},y_{3},z_{3}\right)\mapsto \left(z_{3},{\dot{z}}_{3},{\ddot{z}}_{3}\right)$) and is not observable when *x*_3_ or *y*_3_ ($\eta_{x_{3}^{3}}=\eta_{y_{3}^{3}}=0$) are measured.

When the nodes of the full network are coupled by variable *z* there is a directed path from every vertex to vertex *z*_3_ if *α* ≠ 0. To see that the network observability also depends on the coupling consider when coupling is accomplished via variable *x* (or similarly via variable *y*). The unfolded graph drawn in Fig 5b shows that the resulting network will only be structurally observable if *z*_1_, *z*_2_ and *z*_3_ are simultaneously recorded, even for *α* ≠ 0 and *a*_32_ ≠ 0.

The previous examples help to understand why Gates and Rocha have argued that to represent nodes as variables lacks intrinsic dynamics and that there is often a discrepancy between results related to controllability that only consider the network topology [34].

To summarize, in investigating the observability of a dynamical network, these three ingredients must be considered: i) nodes connected according to an adjacency matrix (a graph), ii) the coupling and iii) the node dynamics.

**Fig 6:**
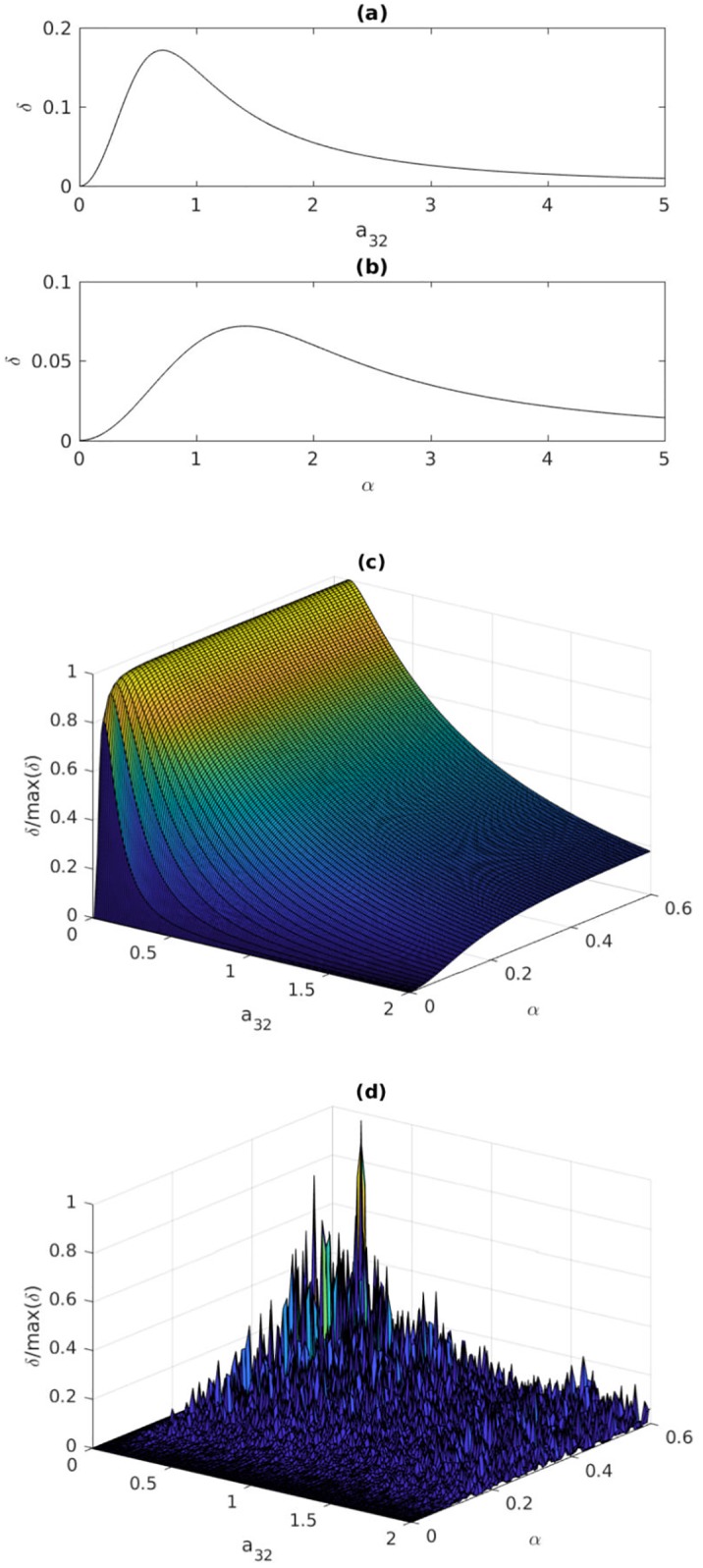
Observability coefficients. Observability coefficient (12) computed (a) for the *network topology* shown in Fig 4a, (b) for the uncoupled *node dynamics* in (24). Mathematically, the network becomes structurally not topologically observable from the sensor node *S*_3_ only for *a*_32_ = 0 and the node dynamics becomes not structurally observable from *z*_3_ only for *α* = 0. Normalized observability coefficients for the *full network* (c) coupled using *z* (Fig 5a), and (d) coupled using *x* (Fig 5b).

Structural topological observability of the full network (Fig 5) is not sensitive to a gradual reduction in observability e.g. due to the decrease of *a*_32_ or *α*. This difficulty can be overcome by quantifying dynamical observability e.g. computing (12) using *A*_adj_ to compose the observability matrix. Dynamical observability of the topology of the network, disregarding the node dynamics, is shown in Fig 6a whereas the dynamical observability of uncoupled node dynamics is shown in Fig 6b. These plots resemble the overall shape of the plots presented in Ref. [7, see their Fig 5].

For the full network in Fig 5a, that is, when three systems (24) are coupled by their variable *z* according to (23) and when *z*_3_ is measured the observability coefficients are shown in Fig 6c. Slices of this plot retain some features of the two previous ones. However, when the nodes are coupled through variable *x* according to the *same* adjacency matrix, the observability is practically lost as illustrated in Fig 6d, where the values in the plot are all close to zero within machine accuracy. This shows that *a joint analysis is required*, that is, not only node dynamics and how the nodes are connected must be used, but also the coupling variables must be taken into account.

An accurate analysis can be performed for a simpler system using $\text{Det}\frac{\partial \Phi_{s}}{\partial x}$. First we set *a*_12_ = *a*_21_ = 0 to treat a two node network {*S*_2_, *S*_3_} with measurements in node *S*_3_, either *x*_3_, *y*_3_ or *z*_3_. We were able to get a full rank 6 × 6 observability matrix with $\Phi_{x_{3}y_{3}z_{3}^{4}}$, $\Phi_{x_{3}^{2}z_{3}^{4}}$ or $\Phi_{y_{3}^{2}z_{3}^{4}}$ for which the determinants were equal to $\pm a_{32}^{3}\alpha^{2}$. The full network becomes structurally non observable when *α*_32_ or *α* is equal to zero as already found. Therefore, the network is observable from *S*_3_ if we measure *z*_3_ plus, at least, another variable from that node.

This simple example shows that investigating a dynamical network by only analyzing the network observability from the adjacency matrix can lead to wrong results because the topological observability is only correct in the extreme case where the nodes are not only coupled but also observed by the variable providing the best observability. If the network is structurally topologically observable, then the network observability depends on the variable with which node dynamics are coupled and observed. Consequently, topological observability must be at least associated with an analysis of the observability of the node dynamics (the isolated system acting at each node) and it must be checked whether the coupling conveys the information up to the measured variable.

## 6 Conclusions

Two decades have past since it was argued that a procedure borrowed from the theory of observability of linear systems could be adapted to explain why global modeling algorithms performed differently using different recorded variables [5]. This paper has aimed at providing a general view of how some concepts related to observability have developed in the realm of nonlinear dynamics and to point out some important differences among the approaches. In order to make distinctions clearer, some different types of observability measures were proposed. Also, the use of the discussed techniques in the field of dynamical networks has been discussed briefly. An overview is provided in Table 1.

An important point to realize is that whereas the definition of observability aims to classify a system as being observable or not, a more interesting challenge is to be able to rank variables *of observable systems* in terms of the potential performance each would have in certain practical situations. The first problem has been connected to structural observability, whereas the second one to dynamical and symbolic observability. These concepts can be readily applied to dynamical systems or to dynamical networks and their three levels of description, namely: node dynamics, topology and the full network.

However, as for the observability of dynamical networks, some limitations of graph-based procedures have been pointed out. It has been argued that the observability of a dynamical network depends on three ingredients: i) the topology described by the adjacency matrix—called topological observability in this paper–; ii) the variable used for coupling nodes and iii) the observability of node dynamics. It was shown that the topological observability of a network—only based on the adjacency matrix—can provide spurious assessment of the observability of the full network in certain cases. In the case of dynamical networks, which are composed of oscillators at the nodes interconnected according to a topology, topological observability does not seem adequate to accurately characterize a network dynamics.
